# Sheathless delivery of a transfemoral pulsatile left ventricular assist device for high-risk percutaneous interventions: a case series

**DOI:** 10.1093/ehjcr/ytaf483

**Published:** 2025-09-29

**Authors:** Edoardo Elia, Marcelo B Bastos, Demarchi Andrea, Giovanni La Malfa, Pistis Gianfranco, Giuseppe Patti, Gabriella Dallaglio, Gioel Gabrio Secco

**Affiliations:** Interventional Cardiology, Azienda Ospedaliero Universitaria SS Antonio e Biagio e Cesare Arrigo, Via Venezia 16, Alessandria 15121, Italy; Department of Interventional Cardiology, Erasmus University Medical Center, Dr Molenwaterplein 40, 3015GD Rotterdam, The Netherlands; Interventional Cardiology, Azienda Ospedaliero Universitaria SS Antonio e Biagio e Cesare Arrigo, Via Venezia 16, Alessandria 15121, Italy; Interventional Cardiology, Azienda Ospedaliero Universitaria SS Antonio e Biagio e Cesare Arrigo, Via Venezia 16, Alessandria 15121, Italy; Division of Cardiology, Azienda Ospedaliero Universitaria SS Antonio e Biagio e Cesare Arrigo, Via Venezia 16, Alessandria 15121, Italy; Department of Translational Medicine, University of Eastern Piedmont, via Solaroli, 17 - 28100 Novara, Italy; Division of Cardiology, University of Parma, Parma I43100, Italy; Interventional Cardiology, Azienda Ospedaliero Universitaria SS Antonio e Biagio e Cesare Arrigo, Via Venezia 16, Alessandria 15121, Italy; Department of Translational Medicine, University of Eastern Piedmont, via Solaroli, 17 - 28100 Novara, Italy

**Keywords:** Percutaneous coronary intervention, Case report, Interventional, Vascular complications, Bleeding, Sheathless, Mechanical circulatory support, iVAC2L, PulseCath

## Abstract

**Background:**

The use of mechanical circulatory support (MCS) can reduce adverse events in patients with severely impaired left ventricular (LV) function and precarious haemodynamics during complex cardiovascular procedures. Despite numerous benefits, MCS requires large-bore access, which increases the risks of vascular complications. The iVAC2L (PulseCath BV, Arnhem, The Netherlands) provides up to 2.0 L/min output using pulsatile flow and is an effective tool for LV unloading, but requires a large-bore sheath (18 Fr).

**Case summary:**

We describe four cases of sheathless insertion of iVAC2L during high-risk cardiovascular interventions. Two cases consisted in high-risk percutaneous coronary interventions, and two other cases were transcatheter edge-to-edge mitral valve repair. All cases were successful, and no adverse events could be observed.

**Discussion:**

The sheathless approach requires an 11% smaller arteriotomy. This case series aims to display how the iVAC2L catheter can be deployed without a sheath to minimize access size in a safe and feasible manner and proposes a structured approach to its deployment. When performed in selected patients, this strategy may reduce vascular complications.

**Conclusion:**

Sheathless introduction of iVAC2L was feasible and safe safely deployed in selected cases of coronary and structural interventions. Larger studies are necessary to further define its impact on event rates.

Learning points

- Percutaneously inserted short-term mechanical support devices have large-bore (>7 Fr) catheter profiles that may hamper the clinical benefits of their use.
- A sheathless approach can reduce arteriotomy profile and improve safety.
- This approach was uneventfully applied in four high-risk cardiovascular interventions, proved to be safe and effective. However, more research is needed to evaluate its impact on safety endpoints.

## Introduction

**Figure 1:**
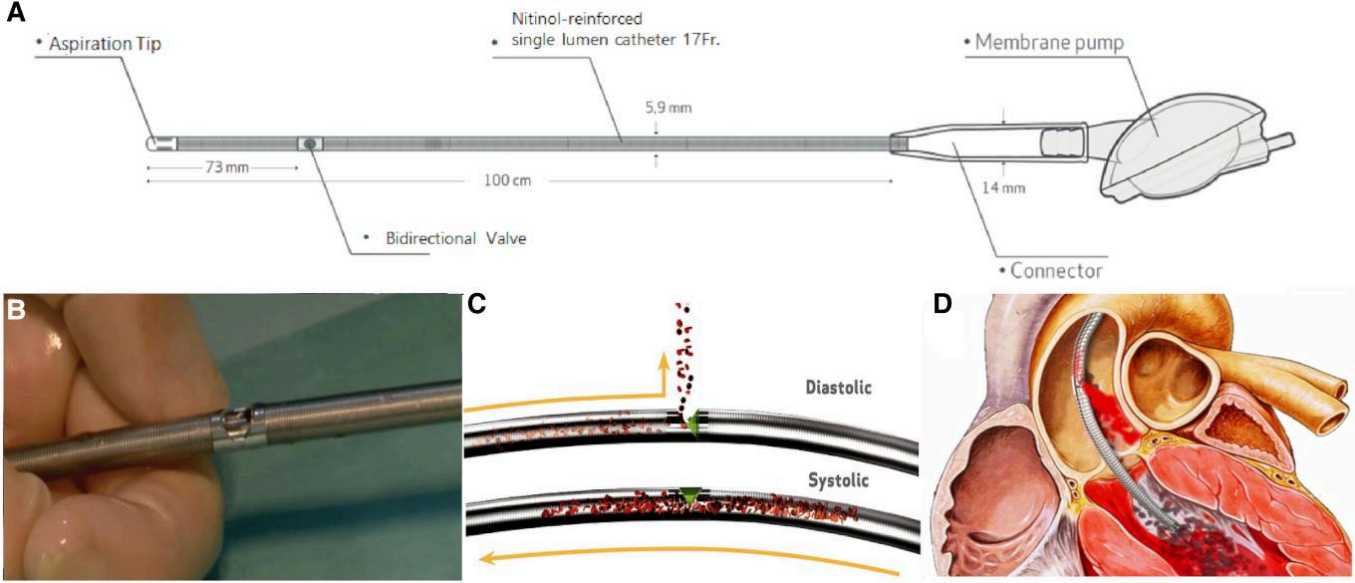
(*A*) The iVAC2L concept. The membrane pump is connected to an intra-aortic balloon pump console, and the inlet tip is positioned inside the left ventricle. The outlet valve, located 73 cm proximal, is placed in the ascending aorta. (*B*) External view of the two-way outlet valve. (*C*) The two different configurations of the two-way valve. In the closed position (systole), blood flows from the inlet tip through the catheter and is collected in the membrane pump. In the open position (diastole), blood is ejected from the membrane pump and is directed to the aortic lumen by the two-way valve. (*D*) Illustrative visualization of the iVAC2L catheter in the correct position across the left ventricular outflow tract.

The adoption of short-term mechanical circulatory support (MCS) devices as a way to reduce adverse events during high-risk cardiovascular interventions is growing. Current evidence favours the use of left ventricular assist devices (LVADs) over intra-aortic balloon pumps (IABPs).^1^ It is supported by randomized and observational studies on elective high-risk percutaneous coronary interventions (HR-PCIs) and acute myocardial infarction with cardiogenic shock (AMICS). These devices are primarily represented by the Impella® (Abiomed, Danvers, MA, USA) and the PulseCath iVAC2L® (PulseCath BV, Arnhem, The Netherlands), among others.^2–6^ The iVAC2L consists of an extracorporeal receptacle divided by a flexible membrane into two separate chambers, one for blood and one for helium. The inlet is positioned in the left ventricle (LV) and the outlet in the ascending aorta (*Figure 1*). Driven by an IABP console, the device aspirates LV blood in systole and ejects it back into the ascending aorta in diastole. The iVAC2L has been shown to effectively unload the LV and to increase the mean arterial pressure (MAP), but has an 18 F large-bore catheter profile due to the use of an insertion sheath.^4–6^

Using large-bore catheters increases the risks of vascular complications, which leads to higher mortality rates, especially among haemodynamically unstable patients.^3,7,8^ A sheathless implantation technique is reportedly able to reduce a device’s footprint.^9,10^ This case series aims to demonstrate the feasibility and safety of a new technique for sheathless iVAC2L insertion.

## Summary figure

**Figure:**
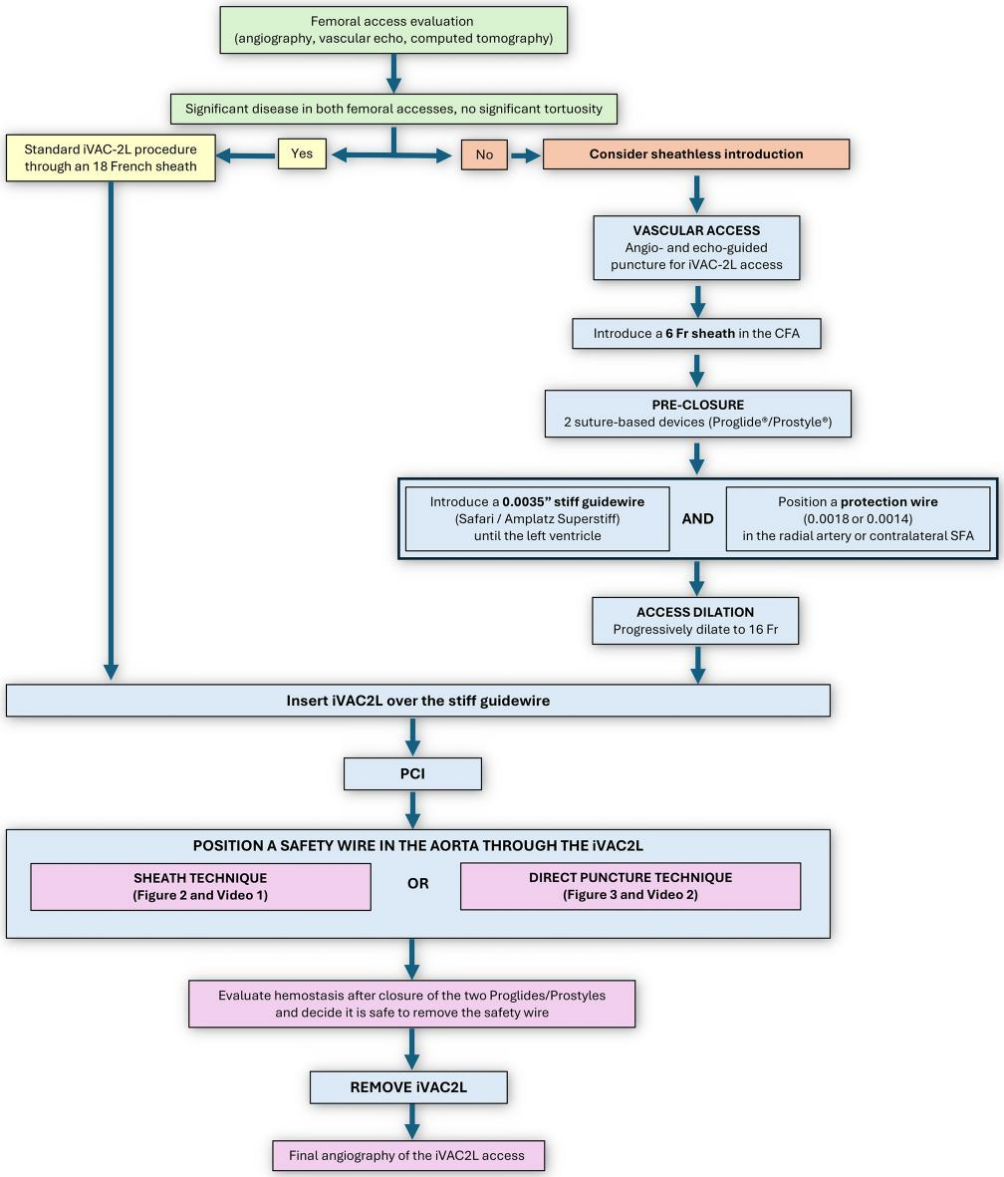
Proposed algorithm to guide the decision to implant iVAC2L with or without a sheath.

## Case descriptions

**Figure 2:**
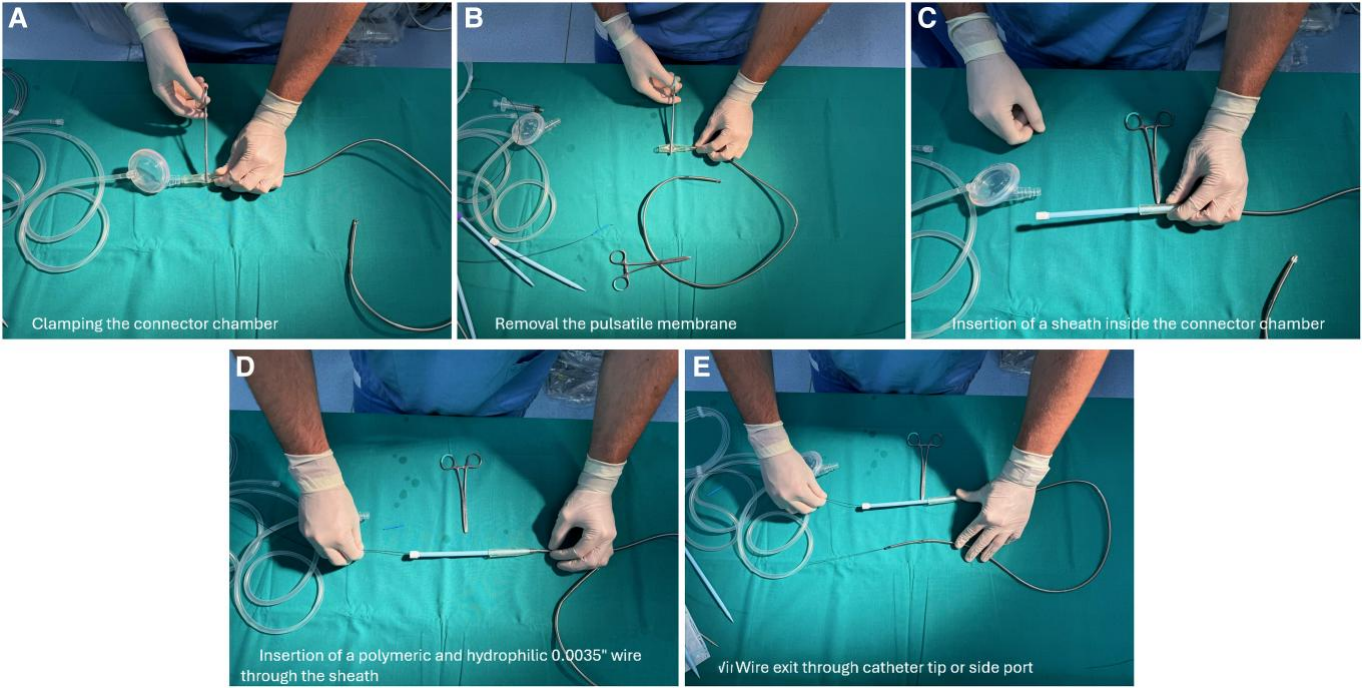
Sheath technique for explantation of iVAC2L. After clamping the connector chamber and removing the pulsatile membrane to prevent blood loss, a 16 Fr sheath is inserted inside the connector chamber. A long polymeric and hydrophilic 0.0035′ wire is then advanced.

A 58-year-old male with a history of hypertension, diabetes, and smoking was admitted to the emergency department for acute decompensated heart failure (ADHF). The patient had recently undergone percutaneous revascularization of the left superficial femoral artery (see Supplementary material online, *Table S1*). The electrocardiogram (ECG) showed reduced R wave progression in precordial leads, and the echocardiogram showed severe LV dilation, with an end-diastolic volume (EDV) of 250 mL and an ejection fraction (EF) of 25%. The right ventricle (RV) had normal dimensions and function, and the aortic valve was normal. Severe functional mitral regurgitation (MR) due to asymmetric tethering, moderate tricuspid regurgitation, dilated vena cava, and severely increased systolic pulmonary pressure (65 mmHg) were also observed. The coronary angiography (CAG) showed a chronic total occlusion (CTO) in the left anterior desscending artery (LAD) and left cirfumflex artery (LCX) and a critical stenosis of the right coronary artery (RCA). The Heart Team opted to perform a PCI of the RCA with drug-eluting stents (DESs) followed by elective treatment of the LAD with MCS. The PCI was performed using angio- and echo-guided punctures of the right radial artery (RA) and of the right and left common femoral arteries (CFA). The iVAC2L was inserted without a sheath in the left CFA after the pre-implantation of two Proglides® and the positioning of a protection wire from the right CFA.^11,12^ Two DESs were successfully deployed in the LAD. Subsequently, a wire was reinserted through the iVAC2L using the sheath technique (*Figure 2*; Supplementary material online, *Video 1*), and the device was removed. The vascular access was closed with the two Proglides®, and the wire was also removed. During the hospital stay, the patient remained haemodynamically stable and successfully underwent up-titration of cardioactive medications, with no reported vascular complications.

**Figure 3:**
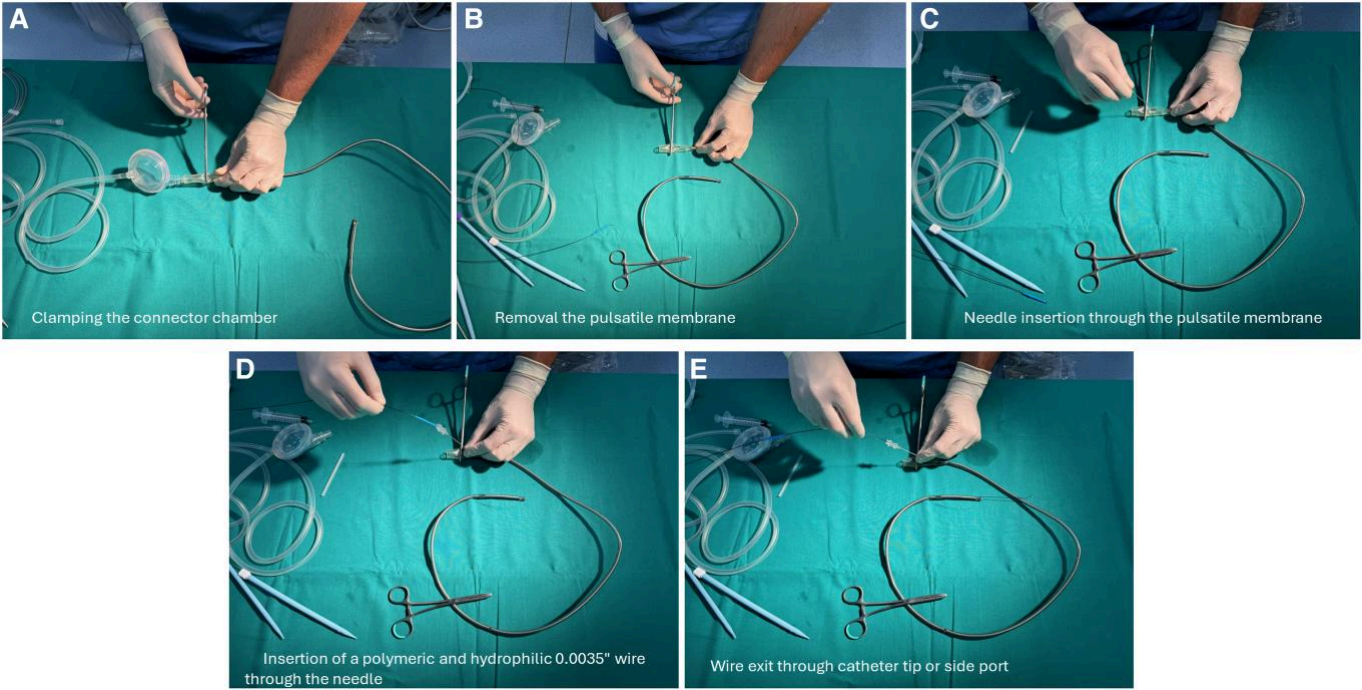
Direct puncture technique for explantation of iVAC2L. The connector chamber is clamped and punctured with a standard femoral access needle, through which a long polymeric and hydrophilic 0.0035′ wire is introduced.

The second patient was a 90-year-old hypertensive male admitted with a non-ST-elevation myocardial infarction (NSTEMI). Echocardiography revealed dilated cardiomyopathy with severe LV dysfunction (EF of 28%) due to global hypokinesia without significant RV dysfunction or abnormal valve disease. The CAG showed CTOs in the RCA and in the ramus intermedius (RI), as well as significant stenoses in the left main artery (LM), in the proximal LCX, and in the proximal and mid-segments of the LAD. Due to the patient’s age and frailty, the Heart Team recommended a percutaneous approach, which was performed from the left CFA. Using echo-guided punctures, the iVAC2L was inserted into the right CFA. Access preparation was performed as previously described. Percutaneous coronary intervention with the ‘Minicrush’ technique was performed in the LM, LAD, LCX, and RCA. Then, the iVAC2L was removed using the direct puncture technique (*Figure 3*; Supplementary material online, *Video 2*). Significant residual bleeding occurred after access closure with two Proglides®, but effective haemostasis was achieved with the addition of a 6 Fr Angioseal®. No vascular access complications were reported during the hospital stay. The patient was discharged 3 days after the procedure and showed initial improvement in the echocardiographic evaluation (EF increased to 39%).

The third patient was a 78-year-old male with a history of nonischaemic dilated cardiomyopathy and permanent atrial fibrillation. He had a biventricular implantable cardioverter-defibrillator (ICD) and had been hospitalized twice in the past year due to ADHF. The patient had previously undergone surgery for prostate cancer and had been diagnosed with haemolytic anaemia as well as moderate to severe kidney disease. He was electively admitted to our department for ADHF.

Echocardiography revealed an EDV of 229 mL, an EF of 32%, and severe MR with coaptation deficit between A2-P2 and A3-P3 (max 4.5 mm). There was also severe atrial dilatation with a left atrial volume index (LAVI) of 55 mL/m^2^. The RV showed no abnormalities. Due to his frailty and comorbidities, the Heart Team decided to perform a transcatheter edge-to-edge repair of the mitral valve (M-TEER) with the MitraClip®. They also decided to use an MCS device to unload the LV and reduce the coaptation gap, thus facilitating the clipping procedure. The iVAC2L was positioned sheathlessly into the right CFA, and a protection wire was placed from the left CFA. The M-TEER was performed through the right common femoral vein (CFV). All access points were acquired through echo-guided puncture. Further aspect of the iVAC2L access preparation and pre-closure were performed as already described in the two previous cases. During the M-TEER procedure, the iVAC2L catheter was retracted above the aortic cusps and kept there to minimize possible interactions between the MitraClip®’s delivery system and the iVAC2L while still providing effective support. This reduced the coaptation gap and enabled effective grasping. After successfully releasing two long and wide (XTW) clips, the MR grade was reduced to mild without significantly increasing the mitral antegrade gradient; and the patient was subsequently transferred to the cardiac intensive care unit. The iVAC2L system is in place to ensure haemodynamic stability. After 8 h with improved haemodynamics, the patient was transferred back to our catheterization laboratory. The iVAC2L was removed after wire reinsertion using the sheath technique (*Figure 2*; Supplementary material online, *Video 1*). Effective closure was achieved using two pre-implanted ProStyles®.^11,12^ During the subsequent hospital stay, no complications were reported. The patient was discharged 7 days later, after receiving levosimendan, with mild MR and an EF of 30%.

**Figure 4:**
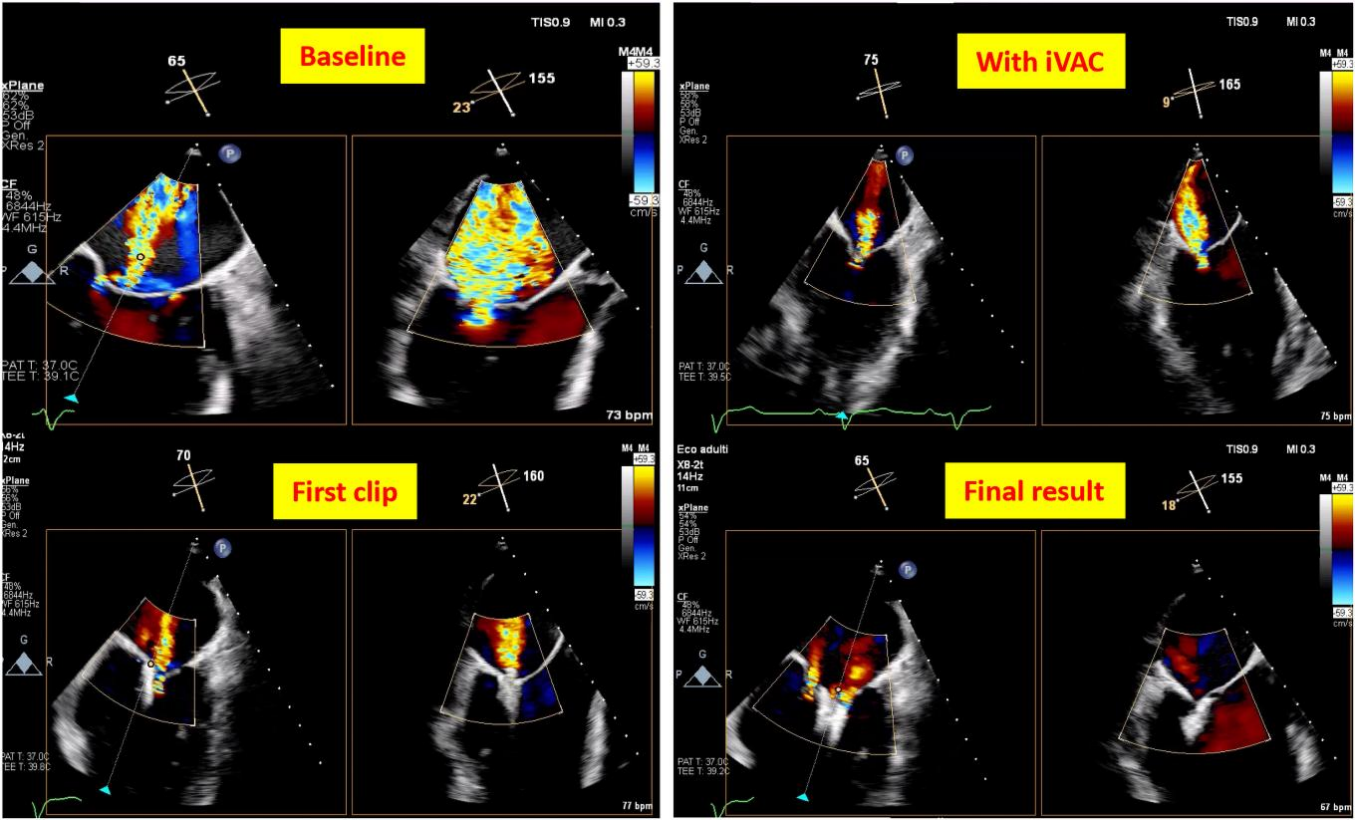
Two-dimensional transesophageal echocardiogram (TEE) with colour flow Doppler during transcatheter edge-to-edge mitral repair showing reduction in the regurgitant jet as the clips are applied and an apparent reduction in LV dimensions with the use of iVAC2L.

The fourth patient was a 79-year-old male with permanent atrial fibrillation, a history of coronary artery bypass grafting (CABG), a bioprosthetic aortic valve, and an ICD. He was admitted for elective M-TEER following multiple episodes of ADHF. Echocardiography revealed severe LV dilation with an EDV of 250 mL and a severely reduced EF of 20%. There was also severe MR due to tethering of both leaflets, severe atrial dilatation (LAVI 51 mL/m²), and a coaptation gap between A2-P2 of 3.2 mm. Transcatheter edge-to-edge repair of the mitral valve with MCS was indicated to achieve the necessary coaptation length. Left superficial femoral artery (SFA) access was obtained through echo-guided puncture with a 6 F sheath to retrogradely advance a 0.0014′ protection wire into the right CFA. Pre-closure was performed as previously described with two ProStyles®.^11,12^ iVAC2L was introduced into the right CFA without a sheath. Transcatheter edge-to-edge repair of the mitral valve was performed through the right CFV. After transseptal puncture and advancement of the clipping system into the LV, the iVAC2L was retracted into the ascending aorta to prevent mechanical interactions. A single XTW clip was implanted, resulting in reduced MR (*Figure 4*). After the procedure, wire reinsertion was done using the direct puncture technique, and the iVAC2L catheter was removed (*Figure 3*; Supplementary material online, *Video 2*). The two pre-implanted ProStyles® were effective in achieving successful access closure and haemostasis. The patient was discharged 3 days later with moderate residual MR; no vascular or haemorrhagic complications were reported.

## Discussion

This is the first report describing the safety of a sheathless iVAC2L deployment. It was performed successfully in two cases of complex high-risk intervention in indicated patients (CHIP)/CTO and two cases of M-TEER. Additionally, the present report brings up new evidence on the efficacy of the iVAC2L in reducing the mitral coaptation gap during M-TEER by means of LV unloading (*Figure 4*, upper right panel) Based on our experience, the sheathless approach can be safely performed in select cases to reduce access size and, consequently, the risk of vascular complications. Consistent with this, no vascular complications or major persistent access-related bleeding occurred.

Despite being a large-bore intervention, iVAC2L has demonstrated a favourable safety profile when applied by experienced practitioners. Recent data shows 30-day rates of 3.4% and 6.9% for major bleeding and major vascular complications, respectively.^4,5^ This bleeding rate is lower than those observed in several large studies involving large-bore interventions, including Impella 2.5 (14 F). This includes the EUROPELLA, USPELLA, and PROTECT I cohorts, as well as the Impella arm of the PROTECT II. However, the same cannot be said for major vascular complications, in which numbers appear to be better than those of structural valve interventions, but still higher than those of the aforementioned studies.^4^ In this context, the sheathless approach used in the present study may indeed be beneficial.

Sheathless insertions are able to reduce the sheath-induced mechanical stretching. Typically, intravascular sheaths are 1–2 Fr larger to accommodate the catheter within. Furthermore, they may facilitate navigation of challenging anatomies (e.g. tortuous).^9,10^ When inserted without a sheath, the iVAC2L requires an arteriotomy that is 11% smaller, which may result in lower rates of vascular complications. However, the efficacy of lowering the arteriotomy profile is most likely conditioned to patient selection.

Selecting patients who may benefit from the use of short-term MCS involves taking steps to prevent vascular complications. Angio- and echo-guided punctures represent our standard practice for selecting an appropriate access site. This approach considers the height of the puncture, the position of the femoral bifurcation, and avoiding unfavourable access spots for suture-based devices (e.g. spotty calcification in the anterior vascular wall). Suture-based devices represent our standard pre-closure technique for vascular accesses > 14 Fr. After the pre-closure devices are in place, the next step is to secure distal protection.

When establishing a large-bore femoral arterial access, it is common practice to preventatively place a 0.0018′ wire or a 0.0014′ stiff wire (e.g. Ironman) in the radial artery or in the contralateral SFA. To provide distal protection, the wire is deployed retrogradely through the aortic bifurcation and positioned distally in relation to the puncture site. There, it secures a prompt bailout pathway to treat any potential access complications at the end of the procedure.

The iVAC2L catheter can be safely explanted by rewiring it prior to its retrieval. This can be done by puncturing the connector chamber (‘direct puncture technique’) or by attaching a sheath to it (‘sheath technique’), as demonstrated in *Figures 2* and *3*. A step-by-step algorithm is also provided in the *Summary figure*; and further details can also be found in the Supplementary material. In our experience, both techniques are equally simple and effective. Finally, standard haemostasis and angiographic control of the access site should be performed post-procedurally as is usually done for large-bore access.

The sheathless approach may be more effective at reducing adverse event rates in patients considered low risk for vascular complications. Since the iVAC2L catheter is soft and has a rounded tip, it may lack the pushability and deliverability required for hostile access anatomies. Therefore, it should always be advanced on a stiff angiographic wire, while the sheathless deployment should be preferably limited to patients with favourable anatomy. This includes patients without significant atherosclerosis or calcification at the access site as well as patients without significant disease or tortuosity in the iliac and femoral arteries.

## Conclusion

This small case series demonstrates that, in select cases, the iVAC2L can be introduced without a sheath to reduce the arteriotomy profile during complex percutaneous interventions. Further investigation of the technique’s feasibility and safety is required through larger studies.

## Lead author biography


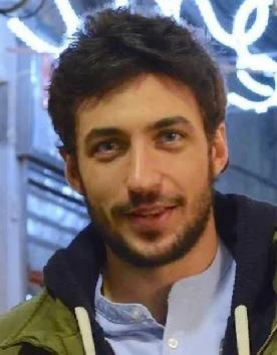


Edoardo Elia is an interventional cardiologist currently working at the Azienda Ospedaliera SS Antonio e Biagio e Cesare Arrigo di Alessandria, Piemonte Orientale, Italy. He concluded a Fellowship in Interventional Cardiology at the Policlinico San Donato (Milan, Italy), residency in cardiology at the Ospedale Policlinico San Martino, and Research Fellowship at the Laboratory of Inflammation and Cardiovascular Disease – University of Genova, Genoa, Italy. He graduated in Medicine at the Università degli Studi di Genova, Italy, in 2018. He has an affiliation with the University of Turim and has contributed in the elaboration of 34 research works in various topics, which cover a broad range of aspects of interventional cardiology.

## Supplementary Material

ytaf483_Supplementary_Data

## Data Availability

The data underlying this article are available in the article.

## Notes

**Consent:** Publication consent has been obtained by all subjects involved in the case series, according to the COPE guidelines.

**Funding:** No specific funding was used to produce this manuscript.
