# Supplementary material for: Sheathless delivery of a transfemoral pulsatile left ventricular assist device for high-risk percutaneous interventions: a case series
Source: Eur Heart J Case Rep. 2025 Sep 29;9(11):ytaf483. doi: 10.1093/ehjcr/ytaf483 (PMC12626125; doi:10.1093/ehjcr/ytaf483)
Supplement: ytaf483_Supplementary_Data [file ytaf483_supplementary_data.zip › Supplement V4 08-09-2025.docx]

Sheathless delivery of a transfemoral pulsatile left ventricular assist device for high-risk percutaneous interventions: a case series

Edoardo Elia MD^1*^; Marcelo B. Bastos MD, MHSc^2*^; Demarchi Andrea, MD^1^; Giovanni La Malfa, MD^1^; Pistis Gianfranco, MD^3^; Giuseppe Patti, MD, PhD^5^; Gabriella Dallaglio, MD^4^; Gioel Gabrio Secco, MD, PhD^1,5^.

1. Interventional Cardiology, Azienda Ospedaliero Universitaria SS Antonio e Biagio e Cesare Arrigo, Alessandria, Italy;
2. Department of Interventional Cardiology, Erasmus Medical Center, Rotterdam, The Netherlands;
3. Division of Cardiology, Azienda Ospedaliero Universitaria SS Antonio e Biagio e Cesare Arrigo, Alessandria, Italy;
4. Division of Cardiology, University of Parma, Parma, Italy;
5. Department of Translational Medicine, University of Eastern Piedmont, Novara, Italy.

Supplement

|  | Patient n° 1 | Patient n° 2 |
| --- | --- | --- |
| Age | 58 years | 90 years |
| Gender | Male | Male |
| Diabetes | Yes | None |
| Hypertension | Yes | Yes |
| Smoking habit | Yes, current | None |
| Dyslipidemia | Yes | Yes |
| COPD | None | Yes |
| PAD | Yes, recent PTA of LSFA | None |
| Previous or current cancer | None | None |
| Atrial Fibrillation | None | None |
| Previous MI | None | None |
| Previous PCI | None | None |
| Previous CABG | None | None |
| Previous cardiac surgery | None | None |
| History of HF | Yes | None |
| Previous PM, ICD or CRT implantation | None | None |
| Admission | ADHF | NSTEMI and ADHF |
| NYHA class at admission | IV | IIb |
| Hemoglobin, g/dL | 13.5 | 14.2 |
| Platelets, 10^3^/mcL | 199 | 186 |
| eGFR (CKD-EPI), mL/min/1,73 | 79 | 54 |
| Troponin peak, ng/L | 409 | 9006 |
| BNP peak, pg/mL | 1016 | 314 |
| LVEF, % | 25 | 28 |
| EDV, ml | 250 | 204 |
| EDD, mm | 66 | 60 |
| Aortic valve disease | Trivial AR | None |
| Mitral valve disease | Moderate MR | Mild-to-moderate MR |
| TAPSE, mm | 18 | 22 |
| Tricuspid valve disease | Moderate TR | Mild-to-moderate TR |
| SPAPs, mmHg | 65 | 37 |
| Type of antiplatelet therapy | Cardioaspirin and Clopidogrel | Cardioaspirin and Ticagrelor |

**Table S1**: Baseline and clinical characteristics of the two patients treated with coronary intervention procedures. COPD: chronic obstructive pulmonary disease; PAD: peripheral artery disease; LSFA: left superficial femoral artery; MI: myocardial infarction; PCI: percutaneous coronary intervention; CABG: coronary artery bypass grafting; HF: heart failure; PM: pacemaker, ICD: implantable cardioverter-defibrillator; CRT-D cardiac resynchronization therapy defibrillator; ADHF: acute decompensated heart failure; NSTEMI: non ST-elevation myocardial infarction; NYHA: New York Heart Association; eGFR: estimated glomerular filtration rate; BNP: B-type natriuretic peptide; LVEF: left ventricular ejection fraction; EDV: end-diastolic volume; EDD: end-diastolic diameter; AR: aortic regurgitation; MR: mitral regurgitation; TAPSE: tricuspid annular plane systolic excursion; TR: tricuspid, SPAP: systolic pulmonary artery pressure.

|  | Patient n° 1 | Patient n° 2 |
| --- | --- | --- |
| Age | 78 years | 79 years |
| Gender | Male | Male |
| Diabetes | None | Yes |
| Hypertension | None | None |
| Smoking habit | Yes, former | None |
| Dyslipidemia | None | None |
| COPD | None | None |
| PAD | None | None |
| Previous or current cancer | None | None |
| Atrial Fibrillation | None | None |
| Previous MI | None | None |
| Previous PCI | None | None |
| Previous CABG | None | Yes |
| Previous cardiac surgery | None | Yes, CABG and aortic valve replacement |
| History of HF | Yes | Yes |
| Previous PM, ICD or CRT implantation | ICD | None |
| Admission | Elective | NSTEMI and ADHF |
| NYHA class at admission | III | IIb |
| Hemoglobin, g/dL | 14.8 | 14.2 |
| Platelets, 10^3^/mcL | 162 | 156 |
| eGFR (CKD-EPI), mL/min/1,73 | 47 | 39 |
| Troponin peak, ng/L | 57 | 132 |
| BNP peak, pg/mL | 1267 | 2066 |
| LVEF, % | 32 | 20 |
| EDV, ml | 229 | 250 |
| EDD, mm | 69 | 72 |
| Aortic valve disease | None | None (normal function of bioprothesis) |
| Mitral valve disease | Severe MR | Severe MR |
| EROa, cmq | 0.56 | 0.39 |
| Coaptation gap | Yes, 4.5 mm | None |
| LAVI, ml/sqm | 55 | 51 |
| TAPSE, mm | 16 | 14 |
| Tricuspid valve disease | Moderate TR | Mild-to-moderate TR |
| SPAPs, mmHg | 58 | 42 |
| Type of antiplatelet therapy | Cardioaspirin | Cardioaspirin |

**Table S2**: baseline and clinical characteristics of patients treated with mitral edge-to-edge repair. COPD: chronic obstructive pulmonary disease; PAD: peripheral artery disease; LSFA: left superficial femoral artery; MI: myocardial infarction; PCI: percutaneous coronary intervention; CABG: coronary artery bypass grafting; HF: heart failure; PM: pacemaker, ICD: implantable cardioverter-defibrillator; CRT-D cardiac resynchronization therapy defibrillator; ADHF: acute decompensated heart failure; NSTEMI: non ST-elevation myocardial infarction; NYHA: New York Heart Association; eGFR: estimated glomerular filtration rate; BNP: B-type natriuretic peptide; LVEF: left ventricular ejection fraction; EDV: end-diastolic volume; EDD: end-diastolic diameter; EROa: effective regurgitant orifice area; LAVI: left atrial volume index; AR: aortic regurgitation; MR: mitral regurgitation; TAPSE: tricuspid annular plane systolic excursion; TR: tricuspid, SPAP: systolic pulmonary artery pressure

|  | Patient n° 1 | Patient n° 2 |
| --- | --- | --- |
| PCI vascular access | Right radial (6Fr) and left femoral (8Fr) | Left femoral (7Fr) |
| iVAC-2L vascular access | Right femoral with contralateral protection | Right femoral with contralateral protection |
| Type of puncture | Echo-guided the femoral PCI access, angio-guided the iVAC-2L access | Echo-guided the PCI access, angio-guided the iVAC-2L access |
| Multivessel disease | Yes, severe stenosis of RCA and CTO of LAD | Yes, severe stenosis of LM, proximal LAD, LCx and Ramus, CTO of RCA proximal |
| Completeness of revascularization | Yes | Yes |
| N° of DES placed | 3 | 4 |
| Bifurcation technique | None | Nanocrush for LM/LAD/LCx bifurcation |
| Length of Support | 38 minutes | 63 minutes |
| Type of vascular closure of iVAC-2L access | Two pre-implanted ProStyles | Two pre-implanted ProStyles and one Angioseal 6Fr for residual bleeding |
| Type of vascular closure of PCI access | One Angioseal 8Fr | One Angioseal 8Fr |

**Table S3:** procedural characteristics of patients treated with coronary intervention PCI: percutaneous coronary intervention; iVAC-2L: ; LM: left main coronary artery; LAD: left anterior descending artery; LCx: left circumflex artery; RCA: right coronary artery.

|  | Patient n° 1 | Patient n° 1 |
| --- | --- | --- |
| M-TEER vascular access | Right femoral vein | Right femoral vein |
| iVAC-2L vascular access | Right common femoral artery with contralateral protection (6Fr) | Right common femoral artery with contralateral protection (6Fr) |
| Type of puncture | Echo-guided for venous access, angio- and echo guided for arterial access | Echo-guided for venous access, angio- and echo guided for arterial access |
| Types of devices implanted | Two XTW | One XTW |
| Length of Support | 78 | 55 |
| Type of vascular closure of iVAC-2L access | Two pre-implanted ProStyles | Two pre-implanted ProStyles |

**Table S4:** procedural characteristics of the two patients treated with transcatheter mitral edge-to-edge repair.

iVAC-2L system percutaneous and sheathless deployment and closure:

Some procedural steps in the sheathless deployment and access closure differ from the traditional technique and are explained below. After obtaining CFA access and inserting a 6F sheath, pre-closure is performed using two suture-based devices (Perclose ProGlide/ProStyle) following the standard pre-closure technique for large-bore access. After pre-closure and aortic valve crossing, a stiff 0.0035" wire (Safari/Amplatz Superstiff) is positioned in the left ventricle (LV), and progressive access dilation is performed up to 16F, followed by the insertion of the iVAC-2L catheter on a stiff angiographic 0.0035” wire. After removing the wire, support can be initiated.

At the end of the procedure, after weaning and interruption of support with the IABP console turned off, the iVAC-2L catheter is retracted into the ascending or descending aorta. At this point, it is crucial to remove the iVAC-2L catheter while maintaining a safety wire inside the vessel to avoid losing position in case of failure of one of the two pre-implanted Proglides/Prostyles or in case of significant residual bleeding. In our center, we perform this in two ways:

- **“Sheath technique”:** After clamping the connector chamber and removing the pulsatile membrane to prevent blood loss, a 16 Fr sheath is inserted inside the connector chamber. A long polymeric and hydrophilic 0.0035" wire is then advanced through it until it exits through the aspiration tip or through a side-port (**Figure 2, Video 1**).
- **“Direct puncture technique”:** After clamping the connector chamber and removing the pulsatile membrane to prevent blood loss, the connector chamber is punctured with a standard femoral access needle. A long polymeric and hydrophilic 0.0035" wire is then advanced through it until it exits through the aspiration tip or through a side-port (**Figure 3, Video 2**).

Afterward, the iVAC-2L catheter is removed, and the access is closed using the standard large-bore access closure technique with the two pre-implanted Proglides/Prostyles. Maintaining a safety wire in position allows for a backup option in case of failure of one of the closure devices, significant residual bleeding, or other complications.

It should be noted that in all cases performed with sheathless iVAC-2L support, access protection was achieved by positioning a wire in the SFA from the contralateral CFA or radial artery and angiography of the access after hemostasis is always performed.
